# Coverage outcomes (effects), costs, cost-effectiveness, and equity of two combinations of long-lasting insecticidal net (LLIN) distribution channels in Kenya: a two-arm study under operational conditions

**DOI:** 10.1186/s12889-020-09846-4

**Published:** 2020-12-07

**Authors:** Eve Worrall, Vincent Were, Agnes Matope, Elvis Gama, Joseph Olewe, Dennis Mwambi, Meghna Desai, Simon Kariuki, Ann M. Buff, Louis W. Niessen

**Affiliations:** 1 Department of Vector Biology, Liverpool School of Tropical Medicine, Pembroke Place, Liverpool, UK; 2 Centre for Applied Health Research and Delivery (CAHRD), Liverpool School of Tropical Medicine, Liverpool, UK; 3 Kenya Medical Research Institute and Centre for Global Health Research, Kisumu, Kenya; 4 Directorate of Quality Management and Digital Health, Ministry of Health and Population, Lilongwe, Malawi; 5 Population Services Kenya (PS Kenya), Nairobi, Kenya; 6 Population Reference Bureau, Nairobi, Kenya; 7 Malaria Branch, Division of Parasitic Diseases and Malaria, Center for Global Health, U.S. Centers for Disease Control and Prevention, Atlanta, GA USA; 8 U.S. President’s Malaria Initiative, Nairobi, Kenya

**Keywords:** Malaria, Vector control, Insecticide-treated nets, Cost-effectiveness, Universal coverage, Kenya, Equity

## Abstract

**Background:**

Malaria-endemic countries distribute long-lasting insecticidal nets (LLINs) through combined channels with ambitious, universal coverage (UC) targets. Kenya has used eight channels with variable results. To inform national decision-makers, this two-arm study compares coverage (effects), costs, cost-effectiveness, and equity of two combinations of LLIN distribution channels in Kenya.

**Methods:**

Two combinations of five delivery channels were compared as ‘intervention’ and ‘control’ arms. The intervention arm comprised four channels: community health volunteer (CHV), antenatal and child health clinics (ANCC), social marketing (SM) and commercial outlets (CO). The control arm consisted of the intervention arm channels except mass campaign (MC) replaced CHV. Primary analysis used random sample household survey data, service-provider costs, and voucher or LLIN distribution data to compare between-arm effects, costs, cost-effectiveness, and equity. Secondary analyses compared costs and equity by channel.

**Results:**

The multiple distribution channels used in both arms of the study achieved high LLIN ownership and use. The intervention arm had significantly lower reported LLIN use the night before the survey (84·8% [95% CI 83·0–86·4%] versus 89·2% [95% CI 87·8–90·5%], *p* < 0·0001), higher unit costs ($10·56 versus $7·17), was less cost-effective ($86·44, 95% range $75·77–$102·77 versus $69·20, 95% range $63·66–$77·23) and more inequitable (Concentration index [C.Ind] = 0·076 [95% CI 0·057 to 0·095 versus C.Ind = 0.049 [95% CI 0·030 to 0·067]) than the control arm. Unit cost per LLIN distributed was lowest for MC ($3·10) followed by CHV ($10·81) with both channels being moderately inequitable in favour of least-poor households.

**Conclusion:**

In line with best practices, the multiple distribution channel model achieved high LLIN ownership and use in this Kenyan study setting. The control-arm combination, which included MC, was the most cost-effective way to increase UC at household level. Mass campaigns, combined with continuous distribution channels, are an effective and cost-effective way to achieve UC in Kenya. The findings are relevant to other countries and donors seeking to optimise LLIN distribution.

**Trial registration:**

The assignment of the intervention was not at the discretion of the investigators; therefore, this study did not require registration.

**Supplementary Information:**

The online version contains supplementary material available at 10.1186/s12889-020-09846-4.

## Background

Despite decades of investment and substantial successes in the scale-up of malaria prevention, treatment and diagnostics throughout malaria endemic countries, malaria remains a significant public health problem. In 2018 it is estimated that 228 million malaria cases and 405,000 malaria deaths occurred with 94% of these being in Africa [1]. While an estimated $2·7 billion was spent globally on malaria control and elimination in 2018, this is less than the $3·2bn spent in 2017, and well short of the estimated $5bn required to achieve the goals set out in the World Health Organizations (WHO) 2016–2030 Global Technical Strategy for Malaria [2]. A substantial share of malaria expenditure comes from international donors and funders, however, endemic country governments are estimated to contribute 30% of total expenditure, making the financing of malaria control a critical concern for both sets of stakeholders [1]. Attainment of the WHO Global Technical Strategy targets will contribute substantially to achievement of the Sustainable Development Goals on health, poverty, equity and sustainable development, underscoring the importance of effective malaria control in delivering a broad development agenda [2].

Long-lasting insecticidal nets (LLINs) are one of two core vector control interventions recommended for universal coverage (UC) by the WHO (the other being indoor residual spraying) [2]. Efforts to scale-up LLIN coverage have had dramatic results. Between 2010 and 2018 the percentage of African households with at least one LLIN increased from 47·8% to 72·0%, and the percentage of the population with access to an LLIN in the household increased from 33·3% to 56·6% [1]. LLIN use has been responsible for two-thirds of the reductions in malaria cases and deaths achieved since 2000 [3, 4], however, in 2018 only 40% of African households had sufficient LLINs for all occupants [1]. Scaling-up delivery of LLINs to achieve and then maintain UC is vital in order to realise the substantial health, economic and development gains that can be achieved from effective malaria prevention.

To achieve and maintain universal LLIN coverage, WHO recommends that countries should apply a combination of distribution through periodic mass campaigns and continuous distribution through multiple channels including antenatal care clinics (ANC) and the expanded programme on immunisation (EPI). While mass campaigns are the only proven way to achieve high and equitable LLIN coverage, continuous channels are essential to fill coverage gaps, account for net deterioration/loss and keep pace with population growth. Tracking the contribution of various delivery channels to overall LLIN coverage is also a key part of monitoring and evaluation efforts, providing vital evidence inform stakeholder decisions on the appropriate combination of delivery channels to scale-up and maintain equitable LLIN coverage with limited resources [5].

The cost of scaling up LLIN coverage is comprised of commodity and distribution costs. Distribution costs account for between 40·5–81·3% of total LLIN programme costs (calculated by authors from data presented in [6]). A recent systematic review found the median cost per net distributed was $4·41 (2016 United States Dollar value) for all distribution channels, however, distribution costs vary between channels with a median cost of $3·87 for mass campaign distribution, $4·69 for continuous distribution channels (including via EPI, ANC, community and school-based channels) and $4·39 for voucher based distribution systems. Interestingly, while the trend in mass campaigns has been one of declining cost per LLIN distributed, continuous health facility-based distribution costs have increased between 2000 and 2016 [7]. Out of 44 separate observations of cost per channel analysed by Wisniewski et al., only five sets of observations represent comparisons of different channels operating in the same country at the same time (concurrent studies). Within these concurrent studies, mass campaign distribution was found to cost less than continuous distribution via ANC in Uganda [8] and less than continuous distribution via ANC and EPI health facilities in Mali [9]; school-based continuous distribution was found to be lower cost than health facility based continuous distribution in both Ghana and Tanzania [9] and ANC based distribution was found to be lower cost than social marketing supported sales in Burkina Faso [10]. Given that costs vary between countries, locations and scale, the relatively small concurrent evidence base is a limitation [10, 11]. A further limitation is the focus on the comparison of individual channels, rather on comparisons of alternative combinations of LLIN distribution channels [12].

LLINs have been demonstrated to be a highly cost-effective malaria control tool in a wide range of settings [6] and while cost per net distributed might be relatively high for some channels, this does not necessarily translate to lower cost-effectiveness. For example, analysis of health facility based continuous distribution channels found that while they cost more than mass campaign distributions, the cost per DALY averted was likely similar for these two channels (due to targeting of vulnerable groups), which were twice as cost-effective as sales/voucher schemes [7]. In addition to cost-effectiveness, LLIN coverage equity remains a key policy concern, and mass campaigns have significantly reduced inequity in household ownership of ≥1 LLIN among wealth quintiles across Africa [13]. The ability of health facility and school-based channels to achieve equitable coverage is a function of equity in access to health and education, which remains a challenge for many countries and the evidence on equity outcomes of community-based delivery strategies is contradictory [14]. As LLIN coverage targets have become more ambitious and distribution channels more numerous, equity must be measured utilising more challenging indicators and targets (i.e. UC as opposed to ≥1 LLIN per house) and there is need to explore the relative contribution of alternative delivery channels or combinations of channels, to achieving equity.

The effectiveness of LLINs in reducing malaria morbidity and mortality is well recognised. However, challenges remain in optimising combinations of delivery channels to scale-up and achieve UC. Addressing cost and equity issues in this effort is critically important. While many studies have evaluated the cost, cost-effectiveness and equity of LLIN distribution channels, few have done so in the same real-life setting and to our knowledge none have attempted to measure the cost-effectiveness of achieving UC or compared alternative combinations of channels. This is the first study to compare cost, effectiveness and equity outcomes of alternative combinations of LLIN distribution channels in a real-life setting. In an era of decreasing donor resources for malaria control and competing priorities for donor and domestic government health spending, the findings of this study will be useful to policy makers and donors in Kenya and other malaria-endemic countries to inform vector control policy and strategy [4].

## Introduction

In 2009, Kenya adopted a LLIN UC strategy in endemic and epidemic-prone areas; the goal was for 80% of people living in areas at-risk for malaria to use LLINs [15]. Sustained malaria control efforts reduced malaria parasitaemia prevalence nationally to 8% among children < 15 years of age by 2014 [16–18]; however, moderate-to-high malaria transmission persists in some areas, including around Lake Victoria in western Kenya, where malaria parasitaemia prevalence was 27% in children < 15 years of age in 2014 [17]. Like most malaria-endemic countries, Kenya has numerous LLIN distribution channels including periodic MCs and continuous distribution through antenatal and child health clinics (ANCC), community health volunteers (CHV), social marketing of subsidised LLINs via rural outlets (SM), commercial for-profit sales of LLINs via retail outlets (CO), and schools [18–22]. Evidence of household coverage estimates and delivery costs (i.e. cost-effectiveness) of these channels is lacking, particularly for multi-channel distribution strategies in real operational settings [10].

This study compares household LLIN ownership and use for two combinations of distribution channels. It also compares total and unit cost, the cost-effectiveness of observed changes in UC, and equity outcomes for the two combinations of channels and the individual channels.

## Methods

### Study design

**Fig. 1:**
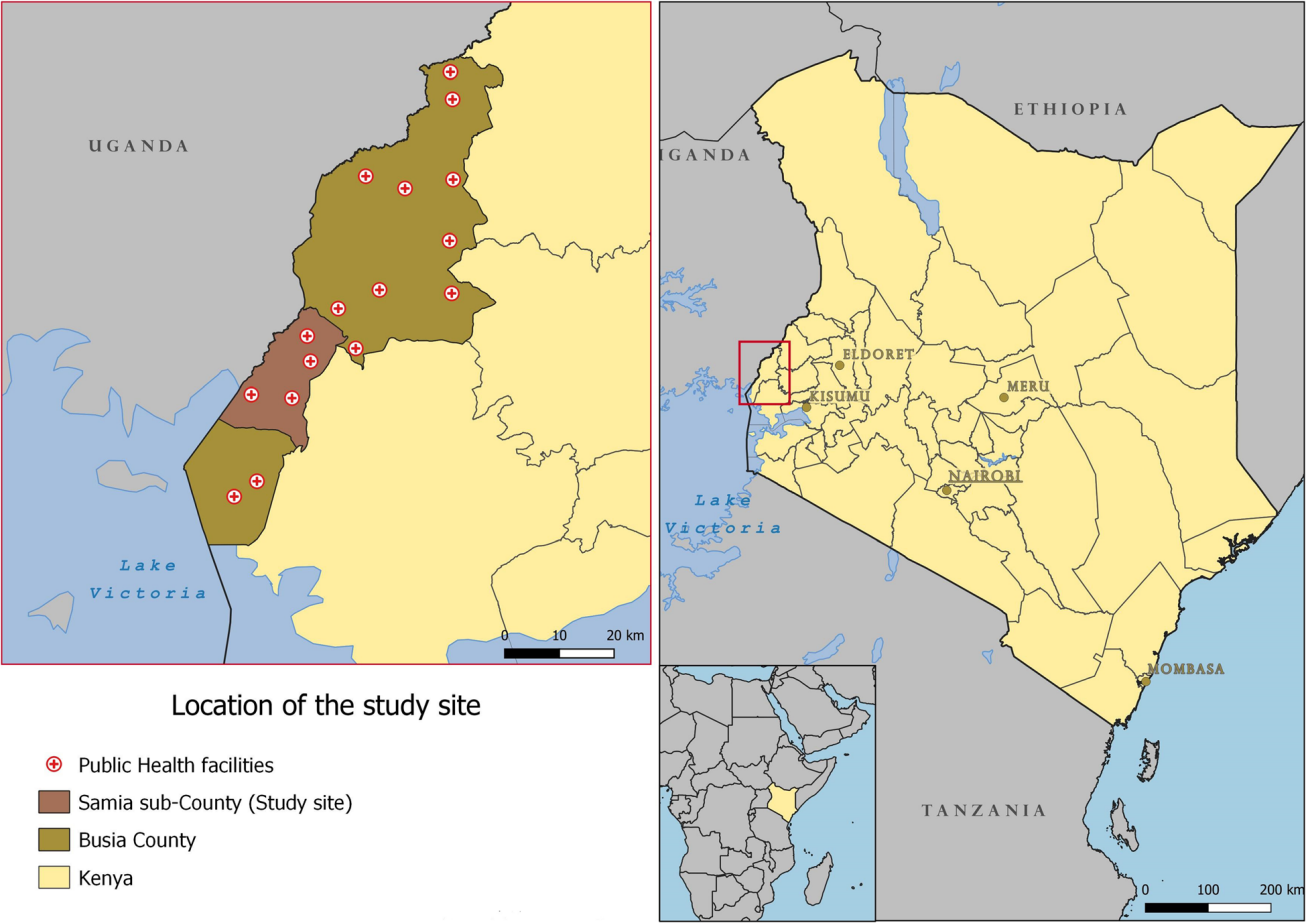
Map of the study area. Image source and rights holder: image produced by and for authors using open source data from, Kenya Master Health Facility List. http://kmhfl.health.go.ke/#/home accessed 22/07/2019

The study was conducted in Samia Sub-county, Busia County in western Kenya (Fig. 1). Samia is largely rural with peri-urban pockets and had an estimated population of 119,246 living in 29 sub-locations (the smallest administrative unit in Kenya) in 2016 [23, 24]. Samia is a malaria-endemic sub-county with 12 public health facilities and an active CHV programme [17]. Kenya implemented a national three-phase MC in 2011–2012, with distribution occurring in Samia in mid-2011 [25]. In 2014, 48·3% of households in Busia County had ≥1 LLIN per two people [26]. A subsequent national MC was conducted between July and November 2015 with distribution in Busia County occurring in October 2015.

The original study design, described fully elsewhere [27], intended to compare the costs and effects (i.e. coverage outcomes) in a real-life setting of a *single* combination of five distribution channels: MC; ante-natal and child health clinics (ANCC); social marketing (SM); commercial outlets (CO) and a pilot continuous LLIN distribution project using community health volunteers to distribute vouchers for nets (CHV LLIN) (Details of each channel are provided in Table S1). The pilot CHV LLIN distribution was initially planned to start directly after the MC to address gaps and maintain UC, with the evaluation planned for 1-year post-MC. Unlike other continuous channels, the CHV channel was (and remains to date) not routinely operational for LLIN distribution in the country. However, two operational factors resulted in modifications to the published study design. First, delays in the MC resulted in CHV distribution preceding the MC. Second, due to insufficient LLINs for the MC, the National Malaria Control Program (NMCP) excluded the area of Samia Sub-county where the CHV channel was operational, from the MC. The CHV channel was implemented in 18 sub-locations (population 63,772) and the MC channel implemented in 11 different sub-locations (population 55,474), with all 29 sub-locations having ANCC, SM and CO. Thus, the modified study design compared *two* combinations of five delivery channels, herein-after referred to as ‘intervention’ and ‘control’ arms. The intervention arm was comprised of four channels: CHV, ANCC, SM and CO, and the control arm was comprised of MC, ANCC, SM and CO.

The CHV channel consisted of two phases: a 12-month *top-up* phase (January to December 2014) to increase the number of LLINs in the household to achieve UC and a 6-month continuous distribution phase (January to June 2015) where UC was monitored and maintained. In both phases, householders received vouchers from CHVs and exchanged vouchers for LLINs at health facilities. The CHV network consisted of 211 CHVs linked to health facilities in the intervention arm areas and 140 CHVs in the control arm areas, however these CHVs were not involved in LLIN distribution and data on the linked facilities in the control area was not available. In each intervention arm sub-location, at least one CHV per village distributed vouchers. The ANCC, CO and SM channels ran continuously throughout the study period.

The primary analysis compared two combinations of the five channels, with the difference between arms being the presence of CHV (intervention) or the presence of MC (control). Secondary analyses explored comparisons between individual distribution channels in the entire study area.

### Effects

As part of the study, a randomised household survey was conducted from July to August 2016 to measure effects (i.e. LLIN ownership, source channel, coverage and use outcomes). Thirty data collectors were recruited and trained by an author (VW) in the use of the survey tool which was pilot tested prior to use. Household heads had the purpose of the study, time implications, risks and benefits and their right to withdraw at any point explained and were given an opportunity to ask questions. Consented heads were asked to sign two copies of the consent form which was witnessed with a copy retained by the household and the study. Data were collected using user-friendly scannable forms developed using TeleForms software. Completed forms were transported to the KEMRI research centre, logged in, scanned and data transferred to the backend MS Access database into password protected desktop computers that were only accessible to the investigators and KEMRI data management personnel. Data codes were developed, and data queries were regularly run for quality control and assurance. Any missing data were resolved by the supervisor as soon as possible. Where households were not available, field workers called back three times until the data was collected. The source channel for each net owned was ascertained during the household survey. Respondents were asked to show the field workers each net in turn and asked, “Where did you get this net from?”. Field workers validated responses based on either known facts about the net sources (e.g. colour and labelling) or channels (e.g. MD nets were green and stickered, ANCC and CHV nets were blue, CHV nets required a voucher or “paper” to obtain). For nets purchased from a shop (CO) or kiosk (SM), field workers were trained to probe for price and specific shop and kiosk details. Field workers validated responses with known prices for subsidised SM nets and SM kiosks details. Field supervisors resolved any uncertainties related to net source in the field.

Reflecting the modifications described above to the protocol, the sample-size for the household survey was increased to 1000 with consenting households selected randomly from community unit registers for the intervention and control arm sub-locations, and data being scanned into the MS Access database, cleaned and analysed in R and Stata [28, 29]. As per protocol, the main effectiveness measure was LLIN use (the proportion of the population that reported sleeping under an LLIN the previous night). The proportions of children < 5 years of age and pregnant women who reported sleeping under an LLIN the previous night, households with ≥1 LLIN and households with UC were also measured. The UC indicator measures the proportion of households that have enough LLINs to cover all individuals who spent the previous night in the surveyed household, assuming one LLIN per two people. Critically, it describes the intra-household coverage gap, i.e. households which own ≥1 LLIN but have not yet achieved UC [30]. In addition to the protocol measures, household LLIN access (proportion of the population that *could* have slept under an LLIN, assuming one LLIN is used by two people) and use of existing LLINs (proportion of existing LLINs reportedly used the previous night) were measured.

Household survey data on the aggregate number and source of LLINs was used to calculate the proportion of LLINs by channel, by arm and for the pooled sample. The proportion of LLINs by channel was also analysed at individual household level by arm and for the pooled sample, with households stratified by coverage level (i.e. households with any net, ≥1 net but not UC, and UC). All indicators were calculated separately for intervention, control arms and pooled sample. Statistical tests for association and difference in proportions were estimated with 95% confidence intervals (CI).

### Cost and cost-effectiveness

Cost data on each channel were retrospectively collected using questionnaires administered to the distribution channel implementers (i.e. Ministry of Health, non-governmental organisation [NGO] partners and commercial outlets). The research team visited all retail shops and wholesalers in the commercial centre of Samia to identify those selling LLINs and request participation and consent. Total economic and financial costs (unannualised and annualised) by channel were calculated in 2015 United States Dollars ($) and summed to compute total costs of all channels operating in the sub-county. Commodity costs (i.e. LLINs) were stripped out of all cost and cost-effectiveness analysis to allow comparison of distribution costs, independent of potential differences in commodity prices.

Unit cost per channel was calculated by dividing the total cost per channel cost by the number of nets or vouchers distributed per channel using data reported by implementing partners (supply-side) and separately, using data on LLIN source/channel as reported in the household survey (demand-side estimate).

**Fig. 2:**
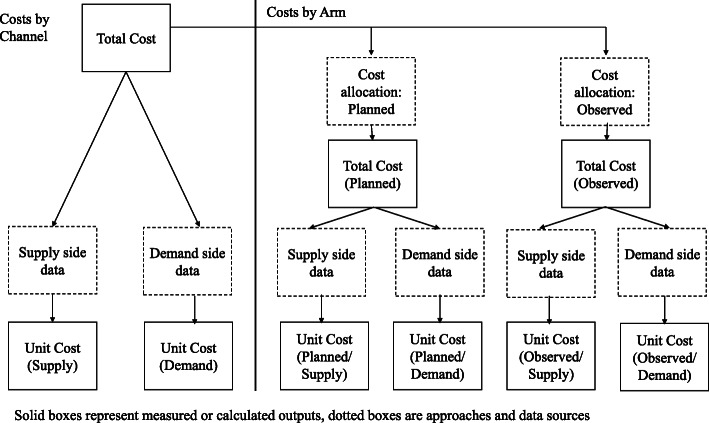
Logic flow chart of the process and methods for calculating by channel and by arm total and unit costs. Source: Authors

Costs were incurred and measured by channel, but for the purpose of the analysis, per channel costs were allocated to arms. Aggregated per channel costs were used to estimate per arm costs in two ways (planned and observed) illustrated by the logic flow chart (Fig. 2). First, based on the trial design, which assumed no MC costs in the intervention arm and no CHV costs in the control arm (planned cost) and with costs of channels operating in both arms split equally. Second, according to the proportion of LLINs by source/channel in the intervention versus control arms from the household survey (observed cost). For example, 201/1279 nets from MC were observed in intervention arm; therefore, 23·5% of MC costs were allocated to intervention arm with the remainder allocated to control arm. Planned and observed cost-per-arm estimates were then used to calculate unit costs per arm by dividing by reported quantity of vouchers/LLINs distributed using supply-side and separately, demand-side data. Supply-side data was the number of vouchers (CHV and SM) or LLINs (MC, ANCC, CO) distributed as reported by the implementers. Demand-side data was the number of nets by LLIN source (channel) for nets recorded in the household survey, multiplied by the sample proportion.

Cost-effectiveness ratios were calculated for each arm and the pooled sample, and the incremental cost-effectiveness ratio (ICER) was calculated to compare the intervention with the control channels. The ICER represents the ratio of change in costs to change in effects and is interpreted using the cost-effectiveness plane. Reflecting global and national UC policy targets and the importance of ownership as a determinant of LLIN use, the cost-effectiveness indicator was cost per additional household with UC (i.e. costs incurred divided by change in UC achieved) calculated for the intervention arm, control arm and the pooled sample. The change in UC was calculated by subtracting the proportion of households with UC at baseline, assuming the same starting point for each arm taken from the 2014 Demographic and Health Survey [26], from the post-intervention proportion of households with UC as measured by the household survey. While the societal perspective is considered gold standard in economic evaluation, decision makers within the health sector may be more interested in the health system perspective which represents resources that they must commit. Hence the analysis was conducted including and excluding the CO channel costs reflecting the difference between societal and health system perspectives, respectively. Uncertainty estimates on cost-effectiveness were obtained by re-running the analysis using the 95% CI range on the proportion of households with UC.

### Equity

Household-level socioeconomic and asset ownership data were used to assign each household to a quintile (1, poorest to 5, least-poor) using principal components analysis [31]. The number and proportion of LLINs per household by quintile were compared by channel, arm and overall. Equity by channel, arm and for the pooled sample (i.e. overall equity) was analysed using concentration indices [32]. A concentration index (C.Ind) gives an indication of equity, independent of contribution to coverage, with a C. Ind of zero implying perfect equity, − 1 the highest degree of pro-poor inequity (distribution favours the poor), and + 1 the highest degree of inequity favouring the least-poor.

## Results

### Household characteristics and coverage

A total of 879 respondents representing unique households (intervention arm 47·8% and control arm 52·2%) were interviewed in July 2016 about all of the LLINs and people in their household. The mean age of respondents was 45·6 years, the majority (70·8%) were female, and 53·9% identified their status as household head. Respondents were similar in sex, mean age and status, in the intervention and control arms, respectively. Most respondents were unemployed (53·5%) or self-employed (42·6%) with intervention arm household heads significantly more likely to be unemployed and significantly less likely to be self-employed than control household heads. Other occupations and education levels were not significantly different between arms (Table 1).

**Table 1:** Study population characteristics

Characteristic	Intervention arm			Control arm			Pooled Sample		
	n	% intervention	% total	n	% control	% total	n	%	Test for association between study intervention and control arm (Chi-squared except^a^), ***p***-value
**Respondent Characteristics (*n*** **= 879)**									
Respondents	420	100·0%	47·8%	459	100·0%	52·2%	879	100·0%	
Male	124	29·5%	48·2%	133	29·0%	51·8%	257	29·2%	0·858
Female	296	70·5%	47·6%	326	71·0%	52·4%	622	70·8%	0·858
Mean age (years)	45·7	–	–	45·5	–	–	45·6		0·9247^a^
Household head	237	56·4%	50·0%	237	51·6%	50·0%	474	53·9%	0·164
**Household Head Occupation (*n*** **= 877, 2 missing)**									
Unemployed	248	59·0%	52·9%	221	48·4%	47·1%	469	53·5%	0·002^b^
Self-employed	155	36·9%	41·4%	219	47·9%	58·6%	374	42·6%	0·001^b^
Government employee	11	2·6%	57·9%	8	1·8%	42·1%	19	2·2%	0·377
Non-government employee	6	1·4%	40·0%	9	2·0%	60·0%	15	1·7%	0·537
**Household Head Education (*n*** **= 871, 8 missing)**									
None completed	63	15·1%	42·6%	85	18·7%	57·4%	148	17·0%	0·165
Primary not completed	172	41·3%	49·4%	176	38·7%	50·6%	348	40·0%	0·423
Primary competed	72	17·3%	46·2%	84	18·5%	53·8%	156	17·9%	0·657
Secondary not completed	44	10·6%	48·4%	47	10·3%	51·6%	91	10·4%	0·905
Secondary completed	41	9·9%	47·7%	45	9·9%	52·3%	86	9·9%	0·986
Vocational	19	4·6%	55·9%	15	3·3%	44·1%	34	3·9%	0·333
University not completed	1	0·2%	100·0%	0	0·0%	0·0%	1	0·1%	0·295
University completed	4	1·0%	57·1%	3	0·7%	42·9%	7	0·8%	0·618

^a^t-test

^b^ significant at 5% level

LLIN use (the proportion of people who reported sleeping under an LLIN the previous night) and UC (proportion of households with at least one LLIN per two people) was significantly lower in the intervention arm compared with the control arm, 84·8% versus 89·2% (*p* < 0·0001) and 76·0% versus 83·9% (*p* = 0·0039), respectively. The proportion of children < 5 years of age sleeping under an LLIN the previous night was lower in the intervention arm (87·6% versus 95·8%, p = 0·0008). LLIN access (persons who could have slept under an LLIN given available number in a household) was also lower in the intervention arm (85·6% versus 89·8%, p < 0·0001). However, the proportion of available LLINs that were reportedly used the previous night was significantly higher in the intervention compared with the control arm (93·3% versus 80·8%, p < 0·0001) (Table 2).

**Table 2:** Long-lasting insecticidal net coverage, access, and use

Indicator	Intervention arm (***n*** = 420 households)		Control arm (***n*** = 459 households)		Pooled sample (n = 879 households)		Test for association between intervention and control arm (Chi squared except^a^), ***p***-value	Difference in Proportions (Control – Intervention) [95% confidence interval for difference]
	n	Proportion [95% confidence interval]	n	Proportion [95% confidence interval]	n	Proportion [95% confidence interval]		
**Households with ≥ 1 LLIN**	**404**	**0·962 [0·939 to 0·977]**	**448**	**0·976 [0·957 to 0·987]**	**852**	**0·969 [0·956 to 0·979]**	**0·2252**	**−0·014 [− 0·037 to 0·009]**
**Households with universal coverage (≥1 LLIN for every two people)**	**307**	**0·76 [0·716 to 0·799]**	**375**	**0·839 [0·802 to 0·87]**	**682**	**0·801 [0·773 to 0·827]**	**0·0039**^**b**^	**−0·079 [− 0·133 to − 0·025]**
Persons with access to an LLIN in their household	1434	0·856 [0·838 to 0·872]	1842	0·898 [0·884 to 0·91]	3276	0·879 [0·868 to 0·889]	< 0·0001^**b**^	−0·042 [− 0·063 to − 0·021]
**Persons who slept under an LLIN the previous night**	**1421**	**0·848 [0·830 to 0·864]**	**1830**	**0·892 [0·878 to 0·905]**	**3251**	**0·872 [0·861 to 0·883]**	**< 0·0001**^**b**^	**−0·044 [− 0·066 to − 0·022]**
Children < 5 years of age who slept under an LLIN the previous night	170	0·876 [0·823 to 0·915]	275	0·958 [0·928 to 0·976]	475	0·983 [0·968 to 0·992]	< 0·0008^**b**^	-0·082 [−0·134 to − 0·030]
Pregnant women who slept under an LLIN the previous night	16	0·889 [0·653 to 0·986]	13	0·929 [0·661 to 0·998]	29	0·906 [0·75 to 0·98]	1·0000 ^a^	-0·04 [−0·270 to 0·219]
LLINs used the previous night	970	0·933 [0·916 to 0·946]	1111	0·808 [0·786 to 0·828]	2081	0·862 [0·847 to 0·875]	< 0·0001^**b**^	0·125 [−0·099 to 0·150]

^a^Small number of counts could lead to errors in p-value; therefore, used Fishers exact test

^b^Significant at 5% level

Indicators explicitly mentioned in the protocol in **bold**, primary per-protocol indictor **bold underlined**, other secondary standard indicators included in the analysis

LLIN Long-lasting insecticidal net

In the pooled sample, 87·2% of the population, 98·3% of children < 5 years of age and 90·6% of pregnant women slept under an LLIN the previous night. At the household level, 96·9% had ≥1 LLIN and 80·1% had UC while individual access to an LLIN was 87·9% and 86·2% of available LLINs were reportedly used (Table 2).

### LLIN numbers and source

There were fewer LLINs in the intervention compared with the control arm (43·4% of total versus 56·6%). Most intervention arm LLINs came from CHV (51·4%), followed by MC (28·1%), ANCC (13·4%), and CO (5·0%). No LLINs were identified as being from the SM channel. Most LLINs in the control arm came from MC (70·0%), followed by CHV (15·3%), ANCC (8·9%), CO and other sources (2·6% each) and SM (0·7%). In the pooled sample, most LLINs were obtained from MC (51·8%) followed by CHV (30·9%), ANCC (10·8%), and CO (3·6%) (Table 3).

**Table 3:** Long-lasting insecticidal nets by source and household coverage strata

Study arm	Mass distribution campaign (MC)	Community Health Volunteer (CHV)	Antenatal and child health clinic (ANCC)	Social Marketing (SM)	Commercial Outlets (CO)	Other^a^	Total
**1. LLINs**							
Intervention	301 (28·1%)	550 (51·4%)	143 (13·4%)	0 (0%)	54 (5·0%)	23 (2·1%)	1071 (43·4%)
Control	978 (70·0%)	214 (15·3%)	124 (8·9%)	10 (0·7%)	36 (2·6%)	36 (2·6%)	1398 (56·6%)
Pooled sample	1279 (51·8%)	764 (30·9%)	267 (10·8%)	10 (0·4%)	90 (3·6%)	59 (2·4%)	2469 (100·0%)
**2. Households with ≥ 1 LLIN/s from each source**^b^							
**a. Any LLIN**							
Intervention	138 (34·2%)	241 (59·7%)	81 (20·0%)	1 (0·2%)	36 (8·9%)	n/a	404
Control	352 (78·7%)	74 (16·6%)	81 (18·1%)	8 (1·8%)	27 (6·0%)	n/a	447^c^
Pooled sample	490 (57·6%)	315 (37·0%)	162 (19·0%)	9 (1·1%)	63 (7·4%)	n/a	851
**b. ≥1 LLIN but not universal coverage**							
Intervention	35 (36·1%)	49 (50·5%)	24 (24·7%)	1 (1·0%)	3 (3·1%)	n/a	97
Control	51 (70·8%)	14 (19·4%)	14 (19·4%)	0 (0%)	3 (4·2%)	n/a	72
Pooled sample	86 (50·9%)	63 (37·3%)	38 (22·5%)	1 (0·6%0)	6 (3·6%)	n/a	169
**c. Universal coverage**							
Intervention	138 (45·0%)	192 (62·5%)	57 (18·6%)	0 (0·0%)	33 (10·7%)	n/a	307
Control	301 (80·3%)	60 (16·0%)	67 (17·9%)	8 (2·1%)	24 (6·4%)	n/a	375
Pooled sample	404 (59·2%)	252 (37·0%)	124 (18·2%)	8 (1·2%)	57 (8·4%)	n/a	682

Underlined text indicates the channel is operating in the arm according to study design

n/a Not applicable

^a^Includes another source (e.g. gift) or the respondent does not know the distribution channel

^b^Households can have nets from multiple sources

^c^LLIN source for one household in this stratum missing, hence total not equal to that in Table 2

Most intervention-arm households with UC obtained ≥1 LLIN from CHV (62·5%), followed by MC (45·0%), ANCC (18·6%), CO (10·7%), and none from SM. Most control-arm households with UC obtained ≥1 LLIN from MC (80·3%), followed by ANCC (17·9%), CHV (16·0%), CO (6·4%), and SM (2·1%). Most pooled sample households with UC obtained ≥1 LLIN from MC (59·2%), followed by CHV (37·0%), ANCC (18·2%), CO (8·4%), and SM (1·2%) (Table 3).

### Cost and cost-effectiveness

The total economic costs (excluding LLIN commodity costs given in Table A1 in the Appendix) of all channels in both arms amounted to $560,049. Supply-side estimates indicate that 71,946 vouchers/LLINs were distributed yielding a unit cost of $7·78 per voucher redeemed/LLIN distributed. Demand-side estimates (household survey) indicate that 64,832 LLINs were distributed, yielding a unit cost estimate of $8·64 per LLIN in household (Table 4).

**Table 4:** Total cost and unit cost per long-lasting insecticidal net or voucher distributed by channel and by arm (US$2015)

	Per Channel Costs						Per Arm Costs				Grand Total (Pooled sample, all channels) or Average
Costs	Mass distribution campaign (MC)	Community Health Volunteer (CHV)	Antenatal and child health clinic (ANCC)	Social Marketing (SM)	Commercial Outlets (CO)	Other^cd^	Costing according to planned by arm allocation (Planned)		Costing according to observed household net ownership by source/channel (Observed)		
							Intervention	Control	Intervention	Control	
**Total Costs**											
Total Economic Cost^a^	104,115·41	216,821·35	195,776·43	24,266·12	19,069·65	no data	336,377·45	223,671·51	296,887·03	263,161·93	560,048·96
Annualised Financial Cost	104,115·41	208,818·43	188,297·34	21,826·34	9259·07	no data	318,509·81	213,806·79	281,233·77	251,082·83	532,316·60
Annualised Economic Cost	104,115·41	208,978·60	188,446·73	21,877·59	9437·18	no data	318,859·35	213,996·16	231,141·45	301,714·05	532,855·51
**Unit Costs**											
(i) **LLINs/vouchers distributed per channel (Supply-side indicator)**											
Reported number	28,870	29,972	8400	4704	no data	no data	36,524^e^	35,422^e^	36,524^e^	35,422^e^	71,946
Economic^a^ Unit Cost	3·61	7·23	23·31	5·16	^b^	^b^	9·21	6·31	8·13	7·43	7·78
Annualised Economic Unit Cost^c^	3·61	6·97	22·43	4·65	^b^	^b^	8·73	6·04	6·33	8·52	7·41
(ii) **LLINs in household (Demand-side indicator)**											
Estimated number	33,584	20,061	7011	263	2363	1549	28,123	36,709	28,123	36,709	64,832
Economic^a^ Unit Cost	3·10	10·81	27·92	92·41	^b^	^b^	11·96	6·09	10·56	7·17	8·64
Annualised Economic Unit Cost^d^	3·10	10·42	26·88	83·32	^b^	^b^	8·22	8·22	10·00	6·84	8·22

^a^Total financial cost equals total economic cost

^b^Cannot be computed as no estimate of LLINs distributed and/or total cost

^c^Supply-side annualised financial costs (not shown) exactly equal to annualised economic costs for all channels except for ANCC $22·42, SM $4·64, Planned: intervention $8·72, Observed: intervention $7·70; control $7·09, and Average $7·40

^d^Demand-side annualised financial unit costs (not shown) exactly equal to annualised economic unit costs except for CHV $10·41, SM $83·12, Planned: intervention $11·33, control $5·82

^e^Assumes 50:50 split of LLINs/vouchers distributed between intervention and control for ANCC and SM

^d^Other sources includes not known or gifts

Total economic costs by arm (excluding LLIN costs) were higher for the intervention arm compared to the control arm using both the planned and observed approaches (planned $336,377 versus $223,671 and observed $296,887 versus $263,162 respectively). Supply-side data indicates that more vouchers/LLINs were distributed in the intervention than control arm 36,525 versus 35,422 yielding higher unit costs per voucher redeemed/LLIN distributed in the intervention than the control arm, regardless of which costing method is applied (planned: $9·21 versus $6·31 and observed: $8·13 versus $7·43, respectively). Demand-side estimates (from household survey data on the total number of nets observed by arm) indicate that fewer LLINs were recorded in the intervention-arm than control-arm households (23,123 compared with 36,709), which also yielded higher unit costs in the intervention compared with the control arm (planned $11·34 versus $5·83 and observed $10·56 versus $7·17, respectively) (Table 4).

Total economic costs by channel (excluding LLIN costs), were highest for CHV ($216,821), followed by ANCC ($195,776), MC ($104,114), and SM ($24,266). Costs were lowest for CO ($19,070), which represented eight outlets (seven retail and one wholesale) identified as selling LLINs. CHVs distributed more vouchers (29,972) than LLINs distributed by the other channels (MC = 28,870, ANCC = 8400, SM = 8400) according to supply-side data. Supply-side estimated unit costs were highest for ANCC ($23·31), followed by CHV ($7·23), SM ($5·16), and MC ($3·61). Demand-side estimates were highest for SM ($92·41), followed by ANCC ($27·92), CHV ($10·81), and MC ($3·10). The unit cost for all channels, except MC, were higher according to demand-side estimates, with the SM channel being almost 18 times higher (Table 4). For per channel cost by activity and health-system level see Supplementary Appendix, Table A2 and A3 respectively.

**Fig. 3:**
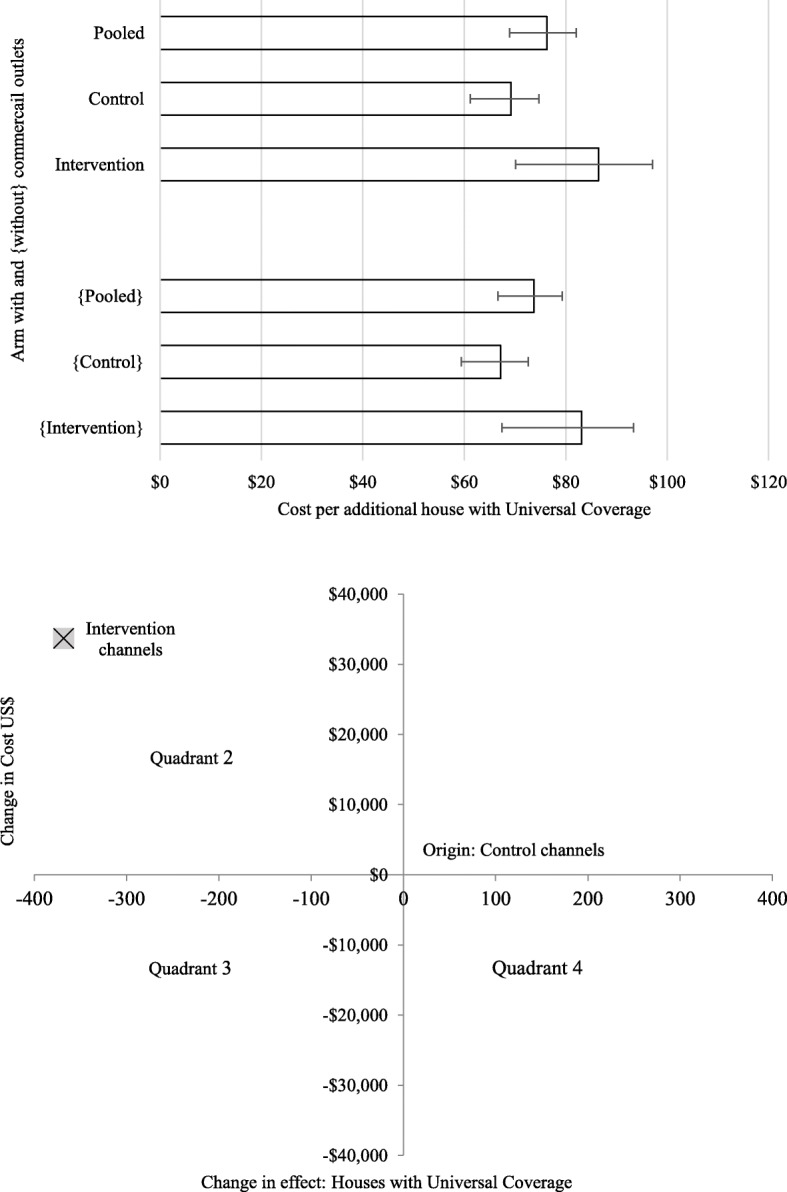
Cost-effectiveness of achieving universal coverage for long-lasting insecticidal nets by study arm. Panel **a** (Top): Cost-effectiveness ratios for intervention, control and pooled sample. Panel **b** (Bottom): Cost-effectiveness plane showing incremental cost-effectiveness ratio (ICER) comparing intervention with control channels. Panel **a**: Error bars show lower and upper bounds of cost-effectiveness calculated using the upper and lower confidence intervals on household long-lasting insecticidal net coverage. Cost-effectiveness excluding CO costs shown in {}. Panel **b**: Origin represents the control channels. Quadrant 1 on the cost-effectiveness plane represents a more effective and more expensive intervention; Quadrant 2 represents a less effective and more expensive intervention; Quadrant 3 represents a less effective and less expensive intervention and Quadrant 4 represents a more effective and less expensive intervention

Pooled sample cost-effectiveness was $76·30 [95% range $70·54–$83·67] meaning that for each additional household achieving UC (compared to pre-distribution baseline), a cost of $76·30 was incurred distributing LLINs or vouchers. Intervention arm cost-effectiveness was less attractive than control although the 95% CIs overlapped ($86·44 [95% range $75·77–$102·77] versus $69·20 [95% range $63·66–$77·23], respectively) (Table 5 and Fig. 3, Panel a). The intervention cost $33,725 more and resulted in 368 fewer additional households with UC than the control. The ICER is -$91·57, which shows that the control is less costly and more effective than the intervention (Fig. 3, Panel b).

**Table 5:** Cost-effectiveness by arm and for the pooled sample from societal and health system perspectives (US$2015)

Cost Indicator and Arm	Study Arm	Marginal Cost-Effectiveness^b^	
		Societal Perspective	Health System Perspective (excludes commercial outlet costs)
**Total Economic Cost**^a^	Intervention	86·44 [75·77–102·77]	83·11 [72·85–98·81]
	Control	69·20 [63·66–77·23]	67·20 [61·81–74·99]
	**Pooled**	**76·30 [70·54–83·67]**	**73·71 [68·13–80·82]**
**Annualised Financial Cost**	Intervention	81·88 [71·78–97·35]	80·27 [70·36–95·42]
	Control	66·03 [60·74–73·68]	65·05 [59·84–72·60]
	**Pooled**	**72·53 [67·04–79·53]**	**71·26 [65·88–78·14]**
**Annualised Economic Cost**	Intervention	67·30 [58·99–80·01]	80·32 [70·41–95·49]
	Control	79·34 [72·98–88·54]	65·10 [59·88–72·65]
	**Pooled**	**72·60 [67·11–79·61]**	**71·31 [65·92–78·20]**

^a^Total financial cost equals total economic cost

^b^Lower and upper bounds of cost-effectiveness calculated using the upper and lower confidence intervals on household long-lasting insecticidal net coverage shown in []

### Equity

**Fig. 4:**
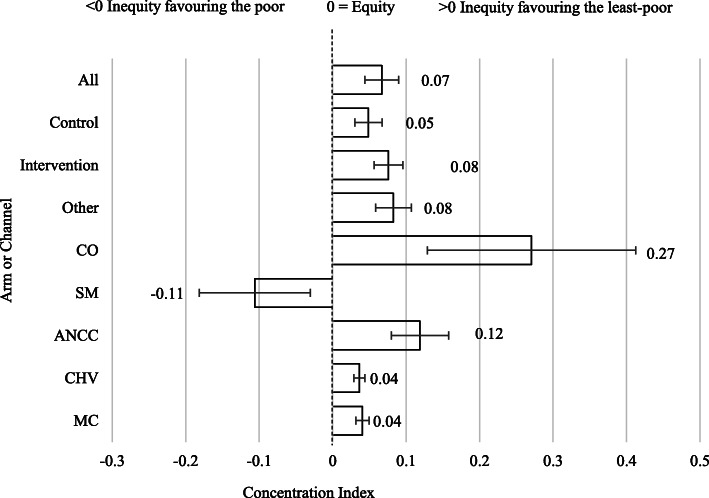
Concentration index of long-lasting insecticidal nets distribution channels, study arm and pooled sample. Error bars indicate 95% confidence interval for concentration index (bar). MC Mass campaign. CHV Community health volunteer. ANCC Antenatal and child health clinics. SM Social marketing of subsidised long-lasting insecticide-treated nets via rural outlets. CO Commercial for-profit sales via retail outlets. Other Sources including not known or gift. All Pooled sample

For all levels of analysis (overall, by channel and by arm) a higher percentage of LLINs were observed in Q5 compared with Q1 (poorest quintile) and in the combined Q4 and Q5 compared with the two poorest quintiles (Q1 and Q2). Overall LLIN distribution was moderately inequitable, favouring the least-poor households (C.Ind = 0·067 [95% CI 0·044–0·090]). The intervention arm C. Ind was higher than that in the control arm, although the difference was not statistically significant (Fig. 4). The per channel C. Ind was closest to zero (i.e. perfect equity) for CHV (C.Ind = 0·036) with MC (C.Ind = 0·041) being the next most equitable channel, although the difference was not statistically significant. ANCC and CO were the least equitable channels (C.Ind = 0·119 and C.Ind = 0·271, respectively). SM was the only channel with a C. Ind below zero (C.Ind = − 0·106) (Table 6).

**Table 6:** Long-lasting insecticidal net distribution by wealth quintile and channel, concentration index and relative concentration index

Comparison	By Channel Comparison^a^												By Arm comparison				Total	
Channel/Arm	Mass Campaign		Community Health Volunteer		Antenatal and Child Health Clinic		Social Marketing		Commercial Outlets		Other		Intervention		Control			
Quintile	n	%	n	%	n	%	n	%	n	%	n	%	n	%	n	%	n	%
**1 (Poorest)**	218	17·1	130	17·0	31	11·6	0	0·0	4	4·4	6	10·2	129	12.0	260	18.6	389	15·8
**2**	251	19·7	155	20·3	52	19·5	1	10·0	11	12·2	12	20·3	234	21.8	248	17.7	482	19·6
**3**	254	19·9	152	19·9	51	19·1	5	50·0	3	3·3	14	23·7	199	18.6	282	20.2	479	19·4
**4**	280	22·0	164	21·5	60	22·5	2	20·0	19	21·1	12	20·3	255	23.8	283	20.2	537	21·8
**5 (Least-poor)**	272	21·3	163	21·3	73	27·3	2	20·0	53	58·9	15	25·4	254	23.7	325	23.2	578	23·4
**All**	**1275**	**100**	**764**	**100**	**267**	**100**	**10**	**100**	**90**	**100**	**59**	**100**	**1071**	**100**	**1398**	**100**	**2465**	**100**
**Concentration Index** **(95% confidence interval)**	0·041 (0·031 to 0·050)		0·036 (0·029 to 0·044)		0.119 (0·080 to 0·158)		-0·106 (-0·181 to 0·030)		0·271 (0·129 to 0·413)		0·083 (0·058 to 0·107)		0·076 (0·057 to 0·095)		0·049 (0·03 to 0·067)		0·067 (0·044 to 0·090)^b^	

^a^Four data points missing for net source by channel

^b^Concentration index for all channel comparison not shown; point estimate is the same as for Total but with a different confidence interval (i.e. C.Ind = 0·067, 95% CI 0·049 to 0·085)

## Discussion

Pooled survey results from a poor, unemployed population with low education status, showed high LLIN ownership, use and access. Between-arm comparisons showed that use (overall and by children < 5 years of age), UC, and access were significantly lower in the intervention-arm versus control-arm areas. These findings are consistent with previous studies showing that the main barrier to LLIN use within households is an insufficient quantity of LLINs [33, 34]. However, the CHV distribution channel resulted in a significantly higher proportion of available LLINs being used in the intervention arm compared to the control arm, which probably represented fewer LLINs in the intervention-arm households. Therefore, a greater proportion of LLINs were in use in the intervention arm.

Fewer LLINs were found in the intervention arm compared with the control arm, suggesting that combinations of channels including MC are more effective at distributing large quantities of LLINs than combinations without MC. The CHV channel was the most important LLIN source overall and for all coverage strata in the intervention arm, and the second most important source overall in the control arm and pooled sample. The MC channel was the most important LLIN source overall and for all coverage strata in the control arm and pooled sample; MC was also the second most important source in the intervention arm. The importance of CHV in the control arm and MC in the intervention arm suggests substantial contamination or movement of LLINs between arms, which could have occurred because of CHV or MC distribution staff or household behaviour. Both LLINs and people are highly mobile.

Our findings on economic cost per LLIN distributed overall ($8·64), by intervention and control arm ($10·56 and $7·17, respectively) are almost double the averages reported in the most recent systematic review, although they are within the range ($4·36, $4·09, $0·86–12·09 mean, median and range respectively [7]). Our results may be on the higher end of the range found in the literature due to them being comprised of a mixture of relatively low (MC) and high (ANCC and SM) cost channels and due to the relatively small quantity of nets distributed which is well below the five million threshold where economies of scale have been found to occur (op cit). By channel, our MC costs are in line with systematic review evidence (supply-side $3·61, demand-side $3·10 compared to $3·82 or $4·09, mean and median respectively) perhaps reflecting the similar nature of MC costs between contexts and over time. Our supply-side estimate of SM costs is higher, though broadly similar, to those in systematic review ($5·16 compared with $4·34 or $3·28 mean and median respectively), however our demand-side estimate ($92·41) is substantially higher. This suggests that some of the SM studies in the literature may be using supply-side estimates, e.g. programmatic data on nets/vouchers sold or distributed, rather than demand-side or household data reflecting ownership or use. ANCC in our study most closely matches the continuous/health facility categorisation in the systematic review, yet our costs are substantially higher ($23·31 and $27·92 supply- and demand-side respectively in our study, compared with $4·63 and $4·22 mean and median respectively in the review. This could reflect the relatively small scale of distribution via these channels, and possibly the upward trend in continuous distribution costs as reported by Wisniewski et al. Their systematic review did not analyse community distribution channel costs separately from other continuous channels, however other studies (included in the review) provide useful comparators. For example, in Democratic Republic of Congo, where community volunteers were used to support a mass campaign strategy the financial cost per net distributed was $2·50 [35] and in Mozambique, LLIN distribution costs were substantially lower at $0·76–0·80 via a community delivery model. Both these costs are substantially lower than our CHV costs ($7·23 and $10·81 supply and demand-side respectively) however, again scale likely plays a role in this as well as methodological differences which may affect comparability i.e. the Mozambique study was a retrospective financial costing which excluded costs above the district level [36]. Note: throughout the discussion costs from the literature have been converted to 2015 US$ by authors for comparison purposes.

Supply-side estimated unit cost and demand-side unit cost were both lowest for MC, which is consistent with the literature. The difference between supply and demand-side unit cost estimates reveals the importance of measuring demand-side outcomes of net distribution channels rather than relying on programmatic data as many studies tend to do. The variation in costs between distribution channels reflects the importance of both scale and delivery channel on unit costs and illustrates the broad range in unit costs for different channels operating in the same setting, with costs of one channel (SM) being over thirty times the unit cost of another channel (MC) in our study. Differences in unit cost may reflect different efficiency levels, economies of scale, or the costs of targeting specific population groups or geographical areas as well as methodological differences between studies.

The marginal cost of each additional household achieving UC was between $63·81–102·77 with the intervention arm being less cost-effective than the control arm, although the difference is not robust to uncertainty. Despite widespread adoption of the UC target, to our knowledge, no other study has attempted to calculate the marginal cost of reaching UC from a baseline existing LLIN coverage or to calculate the ICER comparing two alternative approaches to achieve UC. Hence, we are unable to benchmark these findings. However, economic theory predicts that after a certain point, the costs of increasing coverage will begin to increase; reaching the last person with an LLIN or other public health intervention (e.g. vaccine) is always the costliest, partly explaining why cost per house with UC was substantially higher than unit cost per LLIN distributed.

LLIN distribution was moderately inequitable, resulting in higher ownership in i.e. favouring least-poor households overall and in both the intervention and control arms. SM was the only channel to favour the poorest households (C.Ind less than 1); however, because of the very low number and proportion of LLINs identified from this channel, SM has very little impact on overall equity or coverage. CHV and MC demonstrated relatively low inequity in favour of the least-poor households, with very similar C. Ind values and overlapping confidence intervals. Our results may seem contrary to other studies which have found that MCs reduce inequity [19, 35, 37–39]. However, different baseline levels of coverage and inequity might explain this; it is easier to reduce inequity from a baseline of low coverage and high inequity relative to one of higher coverage and lower inequity. Indeed, other studies have found that community-based distribution can improve equity in ownership of any LLINs but increase inequity of UC [14], suggesting the importance of measuring equity for different, more ambitious coverage indicators.

ANCC and CO both exhibited greater inequity in favour of least-poor households than MC and CHV. However, ANCC is an important LLIN channel for biologically (as opposed to economically) vulnerable people. Although the unit cost of this channel is high, its ability to target biologically vulnerable people (i.e. pregnant women and infants) makes it more cost-effective [7] and its role as a part of the larger package of preventive health services justifies continued policy and financial support for this channel. Similarly, while CO is inequitable, this channel makes a useful contribution to UC without any cost to the health system. SM had the highest unit cost and contributed little to household coverage suggesting less rationale for continuation or expansion of this channel for UC policy objectives.

Conducting evaluation alongside routine implementation is challenging and resulted in deviations from protocol. However, results from real-life evaluations are more informative for policy than research-intervention studies. The high proportion of MC LLINs in the intervention arm and CHV LLINs in the control arm implies contamination (i.e. movement of LLINs) between arms, potentially affecting the study robustness. Additionally, despite extensive efforts to ensure the accuracy of source-channel data during the household survey, respondents might have incorrectly identified or differentiated the LLIN source, particularly between CO and SM channels as both involve shop purchases. Potential recall bias and inaccuracy of supplier reported distribution and cost data are also inherent with this type of study. We addressed these limitations by using both the planned and observed voucher/LLIN distribution as well as both supply- and demand-side estimates of LLINs distributed/vouchers redeemed to calculate and compare the unit costs of different channels and between arms. The comparison of intervention versus control unit costs was the same (i.e. intervention unit cost was higher than control), and the MC channel had the lowest unit costs regardless of the method.

We present the best estimate of per channel and per arm unit costs incurred during the study period. However, we did not seek to assess the extent to which channels were being used to their full capacity, meaning that our results do not necessarily reflect the most efficient (i.e. lowest unit cost) distribution possible. For example, budget-imposed limits on LLIN commodities distributed via each channel may have limited economies of scale. The potential of each channel to handle a higher volume of LLINs would improve both the efficiency and coverage outcomes, which would probably influence the results of relative comparisons between channels. Furthermore, CHV was a new delivery channel being piloted, whereas the others had all been operating for a longer period, hence it is possible that this adversely affected the costs and efficiency of the CHV channel relative to the others.

Kenya’s strategy of a multiple-channel model was successful; in Samia Sub-county, both arms achieved UC and use targets. The combination of channels in the control arm, which included MC, appears to be the most effective way to achieve high coverage and the most cost-effective approach to increase UC. The relatively high cost estimated to reach UC at the household level ($76·30 per house with UC) shows the substantial financial resources required to realistically meet this policy objective. Equity requires use of multiple channels and countries should consider both the scale and equity of individual channels when making policy decisions. Many countries are grappling with how to reach UC targets and increase equity. In comparable rural settings, we expect that other countries could realise similar results using a mix of multiple channels. However, these results might not be generalisable to other settings or to different malaria transmission zones within the same country.

The goal of the original study design was to determine how to *maintain* UC, i.e. measuring the cost and impact on coverage of CHV distribution *in addition* to MC, this remains a critically relevant policy question and further studies on the combination of MC with CHV are urgently needed. However, the original study goal was not possible for the operational reasons outlined above. Therefore, we could not assess the CHV channel contribution to maintaining LLIN coverage post-MC, and the timing of the household survey less than a year after the MC likely affected the study results. Long-term maintenance of LLIN coverage, particularly between mass campaigns, is a key challenge requiring additional research, financial and political commitments. Longitudinal data on LLIN delivery channels and the impact on LLIN coverage, access and use and on malaria prevalence would be useful for countries, donors, and stakeholders.

## Conclusions

In line with best practices, the multiple distribution channel models achieved high LLIN ownership and use in this Kenyan study setting. The control-arm combination, which included MC, was the most cost-effective way to increase UC at household level. Mass campaigns, combined with continuous distribution channels, are an effective and cost-effective way to achieve UC in Kenya. Significant financial resources and distribution efforts will be required to achieve and then maintain UC. The methods used and findings are relevant to other countries and donors seeking to optimise LLIN distribution.

## Supplementary Information

Additional file 1: **Table S1**. Description of Net Distribution Channels Evaluated in Samia, Busia County, Kenya. (DOCX 24 kb)

Additional file 2: Appendix 1. **Table A1**. Reported quantity, purchase price, selling price and calculated gross profit margin per net sold in Kenyan Shillings (KES) and United States Dollars (USD). **Table A2**. Total financial/economic cost (a), annualised financial cost (b) and annualised economic cost (c) by channel and activity ($2015), excluding LLIN commodity cost. **Table A3**. Total financial/economic cost (a), annualised financial cost (b) and, annualised economic cost (c) by channel and level (2015USD), excluding LLIN commodity cost. (DOCX 62 kb)

## Data Availability

The datasets used and/or analysed during the current study are available from the corresponding author on reasonable request.

## Abbreviations

ANCC: Antenatal and child health clinics
CHV: Community health volunteer
CI: Confidence interval
C.Ind: Concentration index
CO: Commercial outlets (shops)
DFID: United Kingdom Government Department for International Development
ICER: Incremental cost-effectiveness ratio
IEC: Information, Education and Communication
KES: Kenyan Shilling
LLIN: Long-lasting insecticidal net
MC: Mass campaign
MoH: Ministry of Health
NGO: Non-governmental organisation
NMCP: National malaria control programme
PMI: United States Government Presidents Malaria Initiative
Q: Quintile (Q1 poorest to Q5 least-poor)
SM: Social marketing
UC: Universal coverage
USD: United states Dollars
