# Supplementary material for: Coverage outcomes (effects), costs, cost-effectiveness, and equity of two combinations of long-lasting insecticidal net (LLIN) distribution channels in Kenya: a two-arm study under operational conditions
Source: BMC Public Health. 2020 Dec 7;20:1870. doi: 10.1186/s12889-020-09846-4 (PMC7720381; doi:10.1186/s12889-020-09846-4)
Supplement: Supplementary file 1 — Table S1. Description of Net Distribution Channels Evaluated in Samia, Busia County, Kenya. (DOCX 24 kb) [file 12889_2020_9846_MOESM1_ESM.docx]

Universal household coverage with insecticide-treated bed nets - efficiency and equity outcomes in malaria-endemic western Kenya

Eve Worrall*, Vincent Were, Agnes Matope, Elvis Gama, Joseph Olewe, Dennis Mwambi, Meghna Desai, Simon Kariuki, Ann M. Buff, Louis W. Niessen

**Table S1 Description of Net Distribution Channels Evaluated in Samia, Busia County, Kenya**

| **Channel** | **Scale and Location** | **Timeframe** | **Responsibility for** | |
| --- | --- | --- | --- | --- |
|  |  |  | **Planning, Financing and Procurement** | **Distribution** |
| Mass distribution campaign (MC) | Nationwide programme: 23 counties. In Samia, MC was carried out in 11 sub-locations referred to as control sub-locations. | Multi-phased UC campaign in 2014-2015.  Mass distribution in Busia County took place during October 2015 and was completed in Samia Sub-county in 1 week. | **Planning, Coordination and Management:** NMCP/MoH*  **Donor Financing:** Global Fund and PMI  **Procurement:** Global Fund-procured LLINs included distribution by supplier to sub-county level. PMI**-**procured LLINs delivered to regional hub, Bungoma. | **Enumeration**  Small teams of local MoH staff and community leaders registered all households and number of persons per household in each area to quantify nets required and alert households to the campaign.  **Information, Education and Communication (IEC)**  Community meetings, local radio announcements, health facilities and other communication channels used to inform the population about household registration and campaign distribution dates.  **Distribution**  An NGO transported PMI-procured nets from the port of entry to Samia. The household head (or representative) came to the designated health facility during the campaign time frame (usually 3−5 days), to collect LLINs. One LLIN per two persons per household were provided free of charge. There was no household level distribution of LLINs during or after the MC. Registered persons who did not come to the health facilities did not receive LLINs. NMCP staff supervised and managed distribution. |
| Distribution via antenatal and child health clinics (ANC) | National: 36 of 47 counties including Busia County. | Ongoing since 2004, when free ITN distribution to pregnant women and children <5 years of age at maternal and child health (MCH) clinics started. | **Planning, Coordination and Management:** NMCP/MoH  **Donor Financing:** DFID and PMI  **Procurement:** LLINs delivered to port of entry | **Distribution**  An NGO transported LLINs to regional warehouse for storage from port of entry. Nets were then distributed to health facilities by NGO; NGO also managed the supply chain in coordination with the NMCP/MoH. Pregnant women and children <1 year of age were given one free LLIN at the first ANC appointment or at the first immunization visit at child health clinics in public and non-for-profit health facilities. All 12 public health facilities in Busia County distributed LLINs via ANC. |

**Table S1 Description of Net Distribution Channels Evaluated in Samia, Busia County, Kenya (continued)**

| **Channel** | **Scale and Location** | **Timeframe** | **Responsibility for** | |
| --- | --- | --- | --- | --- |
|  |  |  | **Planning, Financing and Procurement** | **Distribution** |
| Distribution via community health workers (CHV) | Pilot in Samia sub-county; 18 intervention sub-locations. | Top-up Phase 1:  January-December 2014  Continuous Distribution Phase 2: January-May 2015 | **Planning, Coordination and Management:** NMCP/MoH  **Donor Financing:** PMI  **Procurement:** LLINs delivered to port of entry | **CHV recruitment and training**  An NGO recruited and trained CHVs to assess the need for LLINs at the household level. CHVs were linked to health facilities for reporting purposes and commodity supplies and were supervised by community health extension workers (CHEWs). CHVs do not get a formal salary but receive a modest stipend from the County government or NGO partners *. Each CHV was assigned approximately 50 households for LLIN distribution.  **Net and Voucher Distribution**  An NGO transported LLINs to the regional warehouse for storage from port of entry. The NGO managed transport, storage, and distribution of LLINs to health facilities and managed the voucher process. The LLIN supply chain was managed by the NGO in coordination with the NMCP/MoH.  During the Top-up Phase 1, CHVs visited households to assess how to achieve UC. Damaged nets were repaired with patch kits. Vouchers for new nets were given for additional or replacement nets.  Community members took their voucher(s) to a designated health facility in exchange for free LLIN/s. During the Continuous Distribution Phase 2, CHV monitored households to maintain UC. Vouchers for new nets were given for additional or replacement nets.  Community members took their voucher(s) to a designated health facility in exchange for free LLIN/s. |
| Social Marketing (SM) | Regional:14 counties including all Sub-counties in Busia County. | 2004 to March 2016 Funding officially ended in March 2015, but the program continued through March 2016. | **Planning, Coordination and Management:** NMCP/MoH  **Donor Financing:** DFID  **Procurement:** LLINs delivered to port of entry | **Distribution**  LLINs were procured, transported, stored and distributed to the sellers by an NGO who managed the supply chain in coordination with the NMCP/MOH. Sellers included select rural shops and community-based organizations. In 2015, the target price for a socially-marketed LLIN was approximately $1·50 to the customer.  **IEC**  Rural shops and community-based organizations used community gatherings, local radio and TV shows and advertisements, competitions, drama and theatre to attract customers. |
| Commercial outlets (CO) | Assumed to operate throughout Kenya. | Ongoing | Private commercial retail and wholesalers finance and manage the entire process. There is no public-sector involvement in this distribution system other than via communication campaigns to promote LLIN use.  Commercial for-profit retail outlets stock and sell LLINs at market prices. Prices vary widely based on geographic location, target market, manufacturer, brand, size, material and other factors. Householders visit retail outlets to purchase nets. | |
