# Supplementary material for: Coverage outcomes (effects), costs, cost-effectiveness, and equity of two combinations of long-lasting insecticidal net (LLIN) distribution channels in Kenya: a two-arm study under operational conditions
Source: BMC Public Health. 2020 Dec 7;20:1870. doi: 10.1186/s12889-020-09846-4 (PMC7720381; doi:10.1186/s12889-020-09846-4)
Supplement: Supplementary file 2 — Appendix 1. Table A1. Reported quantity, purchase price, selling price and calculated gross profit margin per net sold in Kenyan Shillings (KES) and United States Dollars (USD). Table A2. Total financial/economic cost (a), annualised financial cost (b) and annualised economic cost (c) by channel and activity ($2015), excluding LLIN commodity cost. Table A3. Total financial/economic cost (a), annualised financial cost (b) and, annualised economic cost (c) by channel and level (2015USD), excluding LLIN commodity cost. (DOCX 62 kb) [file 12889_2020_9846_MOESM2_ESM.docx]

**Appendix 1: Additional costing methods and results (also containing supplementary tables A1 A2 and A3)**

**Supplement to:** Coverage outcomes (effects), costs, cost-effectiveness, and equity of two combinations of long-lasting insecticidal net (LLIN) distribution channels in Kenya: A two-arm study under operational conditions

Brief Title: Universal household coverage with insecticide-treated bed nets - efficiency and equity outcomes in malaria-endemic western Kenya

Eve Worrall*, Vincent Were, Agnes Matope, Elvis Gama, Joseph Olewe, Dennis Mwambi, Meghna Desai, Simon Kariuki, Ann M. Buff, Louis W. Niessen

# **Methods**

Provider cost data were collected retrospectively, per protocol, using questionnaires, reviews of expenditure data and meetings with donors, partners and the NMCP/MoH. Cost data was entered into Excel spreadsheets, costs in Kenyan Shillings (KES) were converted to United States Dollars (USD) using the average annual exchange rate for 2015. Costs incurred for activities in more than one county (e.g., central-level support), which could not be assigned using the micro-costing (i.e. bottom up) approach, were allocated to Samia Sub-county using a suitable proxy. To facilitate comparison of costs across the different distribution channels, a set of standard activities were developed to capture key activities for all distribution channels (e.g. administration, coordination and management; communication and mobilisation; net storage and distribution; training). All costs were then allocated to one of these categories independently by two authors (EW and VW) with each discrepancy discussed and resolved. Likewise, each item was categorised by the level at which the cost was incurred (i.e. national, regional, sub-county or sub-location) independently by two authors (EW and VW) and each discrepancy discussed and resolved.

Since the analysis is focussed on LLIN distribution costs and the cost of LLINs procured differed between channels, LLIN costs were excluded from the main analysis, instead, the purchase price, brand and quantity of nets purchased by channel are presented in this Appendix.

As per protocol, the total cost and cost by channel are presented as financial and economic costs. In addition, annualised total and unit costs are presented. Annualised financial costs are calculated by dividing capital costs (i.e. assets with a useful life of more than one year) by their useful life, and annualised economic costs were calculated the same and discounted at 3%. All costs are presented by channel, activity and health system level with costs expressed as a number and percentage of total costs to identify cost drivers and differences between channels.

# **Results**

# **Net prices**

Distribution channel implementer questionnaires revealed that MD, CHV and ANC channels paid the same price for LLINs ($4·07). Among the commercial outlets, reported purchase price ranged from $1·02 to $3·56, with selling price and gross profit margin per net sold ranging from $2·55 to $5·09 and $0·51 to $2·55, respectively (Table A1).

# **Table A1 Reported quantity, purchase price, selling price and calculated gross profit margin per net sold in Kenyan Shillings (KES) and United States Dollars (USD)**

| Channel and Outlet (for CO) | Net Brand | Reported purchase price | | Reported selling price | | Gross margin (US$) |
| --- | --- | --- | --- | --- | --- | --- |
|  |  | KES | US$ | KSH | US$ |  |
| MD | Permanet | 400 | 4·07 | 0.00 | 0.00 | -4.07 |
| CHV | Permanet | 400 | 4·07 | 0·00 | 0·00 | -4·07 |
| ANC | Unstated | 400 | 4·07 | 0·00 | 0·00 | -4·07 |
| SM | Supanet xtra power (Olyset Brand) | no data | no data | no data | no data | no data |
| CO1 | Meng Mei | 250 | 2·55 | 400·00 | 4·07 | 1·53 |
| CO2 | Romantic house | 350 | 3·56 | 450·00 | 4·58 | 1·02 |
| CO3 | SafiNet | 150 | 1·53 | 250·00 | 2·55 | 1·02 |
| CO4 | SafiNet | 450 | 4·58 | 500·00 | 5·09 | 0·51 |
| CO5* | SafiNet | 150 | 1·53 | 250·00 | 2·55 | 1·02 |
| CO6 | SafiNet | 300 | 3·06 | 450·00 | 4·58 | 1·53 |
| CO7 | Supernet | 250 | 2·55 | 350·00 | 3·56 | 1·02 |
| CO8 | SupernetExtra | 100 | 1·02 | 350·00 | 3·56 | 2·55 |

Each CO number represents an individual shop· *CO5 was reported as being a wholesaler

Data source: Distribution channel implementer questionnaires

# **Costs**

By activity (Table A2) personnel costs were the highest contribution to costs in ANC and CO, whereas distribution and transport were the biggest drivers in MD and SM. Training and meeting costs were the biggest cost driver in the CHV channel. By level, (Table A3) the majority of costs are incurred at the sub-Location level in the MD, ANC and CHV channels. All costs are incurred at sub-Location level (i.e. individual shop) for the CO channel. SM costs are heavily skewed (80%) to the national level.

# **<INSERT TABLE A2 HERE>**

# **<INSERT TABLE A3 HERE>**

# **Discussion**

Annualised economic (and financial) costs are very similar to total costs due to the low proportion of capital costs in all channels. Economic and financial costs are also very similar and because no donated resources were identified in the costing the difference is entirely down to discounting of capital costs in the economic analysis. Full details of unit costs and quantities of all resources (including bed net costs excluded from the costing) by distribution channels are available from the authors on request.

# **Table A2 Total financial/economic cost (a), annualised financial cost (b) and annualised economic cost (c) by channel and activity ($2015), excluding LLIN commodity cost**

| **Cost category** | **Admin, Coordination and Management** | | **Buildings** | | **Communication and Mobilisation** | | **Distribution and Transport** | | **Monitoring and Evaluation** | | **Storage** | | **Training** | | **Total Cost** |
| --- | --- | --- | --- | --- | --- | --- | --- | --- | --- | --- | --- | --- | --- | --- | --- |
| **a. Total financial/economic cost and %** | | | | | | | | | | | | | | | |
| **MD** | 31,252 | 30·0% | 0 | 0% | 6,576 | 6·3% | 51,402 | 49·4% | 0 | 0% | 7,501 | 7·2% | 7,385 | 7·1% | 104,115 |
| **CHV** | 14,818 | 6·8% | 0 | 0% | 23,121 | 10·7% | 30,110 | 13·9% | 0 | 0% | 4,889 | 2·3% | 143,884 | 66·4% | 216,821 |
| **ANC** | 97,154 | 49·6% | 0 | 0% | 0 | 0% | 81,822 | 41·8% | 11,819 | 6·0% | 4,981 | 2·5% | 0 | 0% | 195,776 |
| **SM** | 5,740 | 23·7% | 0 | 0% | 6,481 | 26·7% | 9,545 | 39·3% | 0 | 0% | 2,369 | 9·8% | 130 | 0·5% | 24,266 |
| **CO** | 6,009 | 31·5% | 10,219 | 53·6% | 70 | 0·4% | 1,840 | 9·6% | 0 | 0% | 931 | 4·9% | 0 | 0% | 19,070 |
| **Grand Total** | 154,973 | 27·7% | 10,219 | 1·8% | 36,248 | 6·5% | 174,719 | 31·2% | 11,819 | 2·1% | 20,671 | 3·7% | 151,399 | 27·0% | 560,049 |
| **b. Annualised financial cost and %** | | | | | | | | | | | | | | | |
| **MD** | 31,252 | 30·0% | 0 | 0% | 6,576 | 6·3% | 51,402 | 49·4% | 0 | 0% | 7,501 | 7·2% | 7,385 | 7·1% | 104,115 |
| **CHV** | 13,944 | 6·7% | 0 | 0% | 23,121 | 11·1% | 22,980 | 11·0% | 0 | 0% | 4,889 | 2·3% | 143,884 | 68·9% | 208,818 |
| **ANC** | 96,805 | 51·4% | 0 | 0% | 0 | 0% | 74,693 | 39·7% | 11,819 | 6·3% | 4,981 | 2·6% | 0 | 0% | 188,297 |
| **SM** | 5,402 | 24·7% | 0 | 0% | 6,481 | 29·7% | 7,444 | 34·1% | 0 | 0% | 2,369 | 10·9% | 130 | 0·6% | 21,826 |
| **CO** | 6,009 | 64·9% | 409 | 4·4% | 70 | 0·8% | 1,840 | 19·9% | 0 | 0% | 931 | 10·1% | 0 | 0% | 9,259 |
| **Grand Total** | 153,413 | 28·8% | 409 | 0·1% | 36,248 | 6·8% | 158,358 | 29·7% | 11,819 | 2·2% | 20,671 | 3·9% | 151,399 | 28·4% | 532,317 |
| **c. Annualised economic cost and %** | | | | | | | | | | | | | | | |
| **MD** | 31,252 | 30·0% | 0 | 0% | 6,576 | 6·3% | 51,402 | 49·4% | 0 | 0% | 7,501 | 7·2% | 7,385 | 7·1% | 104,115 |
| **CHV** | 13,962 | 6·7% | 0 | 0% | 23,121 | 11·1% | 23,122 | 11·1% | 0 | 0% | 4,889 | 2·3% | 143,884 | 68·9% | 208,979 |
| **ANC** | 96,812 | 51·4% | 0 | 0% | 0 | 0% | 74,835 | 39·7% | 11,819 | 6·3% | 4,981 | 2·6% | 0 | 0% | 188,447 |
| **SM** | 5,411 | 24·7% | 0 | 0% | 6,481 | 29·6% | 7,486 | 34·2% | 0 | 0% | 2,369 | 10·8% | 130 | 0·6% | 21,878 |
| **CO** | 6,009 | 63·7% | 587 | 6·2% | 70 | 0·7% | 1,840 | 19·5% | 0 | 0% | 931 | 9·9% | 0 | 0% | 9,437 |
| **Grand Total** | 153,447 | 28·8% | 587 | 0·1% | 36,248 | 6·8% | 158,684 | 29·8% | 11,819 | 2·2% | 20,671 | 3·9% | 151,399 | 28·4% | 532,856 |

#

# **Table A3 Total financial/economic cost (a), annualised financial cost (b) and, annualised economic cost (c) by channel and level (2015USD), excluding LLIN commodity cost**

| **Health system level where cost incurred** | **National** | | **Regional** | | **Sub-County** | | **Sub-Location** | | **Total Cost** |
| --- | --- | --- | --- | --- | --- | --- | --- | --- | --- |
| **a. Total financial/economic cost and %** | | | | | | | | | |
| MD | 27,331 | 26·3% | 7,405 | 7·1% | 27,297 | 26·2% | 42,083 | 40·4% | 104,115 |
| ANC | 55,704 | 28·5% | 0 | 0% | 67,987 | 34·7% | 72,085 | 36·8% | 195,776 |
| CHV | 43,410 | 20·0% | 23,848 | 11·0% | 10,846 | 5·0% | 138,717 | 64·0% | 216,821 |
| SM | 19,547 | 80·6% | 3 | 0·0% | 3,307 | 13·6% | 1,409 | 5·8% | 24,266 |
| CO | 0 | 0·0% | 0 | 0% | 0 | 0% | 19,070 | 100·0% | 19,070 |
| **Grand Total** | 145,992 | 26·1% | 31,256 | 5·6% | 109,437 | 19·5% | 273,364 | 48·8% | 560,049 |
| **b. Annualised financial cost and %** | | | | | | | | | |
| MD | 27,331 | 26·3% | 7,405 | 7·1% | 27,297 | 26·2% | 42,083 | 40·4% | 104,115 |
| ANC | 48,225 | 25·6% | 0 | 0% | 67,987 | 36·1% | 72,085 | 38·3% | 188,297 |
| CHV | 36,280 | 17·4% | 22,975 | 11·0% | 10,846 | 5·2% | 138,717 | 66·4% | 208,818 |
| SM | 17,495 | 80·2% | 3 | 0·0% | 2,920 | 13·4% | 1,409 | 6·5% | 21,826 |
| CO | 0 | 0% | 0 | 0% | 0 | 0% | 9,259 | 100·0% | 9,259 |
| **Grand Total** | 129,331 | 24·3% | 30,383 | 5·7% | 109,050 | 20·5% | 263,553 | 49·5% | 532,317 |
| **c. Annualised economic cost and %** | | | | | | | | | |
| MD | 27,331 | 26·3% | 7,405 | 7·1% | 27,297 | 26·2% | 42,083 | 40·4% | 104,115 |
| ANC | 48,374 | 25·7% | 0 | 0% | 67,987 | 36·1% | 72,085 | 38·3% | 188,447 |
| CHV | 36,422 | 17·4% | 22,993 | 11·0% | 10,846 | 5·2% | 138,717 | 66·4% | 208,979 |
| SM | 17,536 | 80·2% | 3 | 0·0% | 2,930 | 13·4% | 1,409 | 6·4% | 21,878 |
| CO | 0 | 0% | 0 | 0% | 0 | 0% | 9,437 | 100·0% | 9,437 |
| **Grand Total** | 129,663 | 24·3% | 30,401 | 5·7% | 109,060 | 20·5% | 263,731 | 49·5% | 532,856 |
